# Supplementary material for: A statistical approach for identifying primary substrates of ZSWIM8-mediated microRNA degradation in small-RNA sequencing data
Source: BMC Bioinformatics. 2023 May 12;24:195. doi: 10.1186/s12859-023-05306-z (PMC10176919; doi:10.1186/s12859-023-05306-z)
Supplement: Supplementary file 1 — Additional file 1: Table S1. miRNAs in the Shi et al. datasets identified as ZSWIM8-sensitive. Table S2. Raw initial parameter values for BBUM model fitting. Figure S1. Application of the BBUM correction method to RNA-seq datasets measuring the effects of transfecting a miRNA into either HeLa or HEK293FT cells. A Plots of log2 fold changes in mRNA levels observed upon transfection of the indicated miRNA duplexes, showing all 29 datasets analyzed. Colors are as in Figure 5. B Volcano plots of raw p values as a function of mRNA log2 fold change, showing all 29 datasets analyzed. Colors are as in Figure 5 [file 12859_2023_5306_MOESM1_ESM.docx]

# Additional file 1

**Table S1.** miRNAs in the Shi et al. datasets identified as ZSWIM8-sensitive.

| Cell line | miRNA | Notes |
| --- | --- | --- |
| *Not changed* | | |
| S2 | dme-miR-7-5p |  |
| A549 | hsa-miR-7-5p |  |
| HeLa | hsa-miR-7-5p |  |
| K562 (KD, A) | hsa-miR-7-5p |  |
| K562 (KD, B) | hsa-miR-7-5p |  |
| MCF-7 | hsa-miR-7-5p |  |
| iMN | mmu-miR-7a-5p |  |
| MEF | mmu-miR-7a-5p |  |
| iMN | mmu-miR-7b-5p |  |
| S2 | dme-miR-9b-5p |  |
| S2 | dme-miR-9c-5p |  |
| S2 | dme-miR-12-5p |  |
| MEF | mmu-miR-17-5p |  |
| MEF | mmu-miR-20a-5p |  |
| HeLa | hsa-miR-29b-3p |  |
| iMN | mmu-miR-29b-3p |  |
| MEF | mmu-miR-29b-3p |  |
| iMN | mmu-miR-33-5p |  |
| MEF | mmu-miR-33-5p |  |
| MEF | mmu-miR-92a-3p |  |
| MEF | mmu-miR-93-5p |  |
| MCF-7 | hsa-miR-145-5p |  |
| A549 | hsa-miR-154-3p |  |
| HeLa | hsa-miR-154-3p |  |
| iMN | mmu-miR-154-3p |  |
| MEF | mmu-miR-154-3p |  |
| MEF | mmu-miR-181b-5p |  |
| S2 | dme-miR-190-5p |  |
| MEF | mmu-miR-193a-3p |  |
| MEF | mmu-miR-195a-5p |  |
| S2 | dme-miR-277-3p |  |
| S2 | dme-miR-279-3p |  |
| MEF | mmu-miR-322-5p |  |
| HeLa | hsa-miR-335-3p |  |
| MCF-7 | hsa-miR-335-3p |  |
| iMN | mmu-miR-335-3p |  |
| MEF | mmu-miR-335-3p |  |
| iMN | mmu-miR-341-3p |  |
| A549 | hsa-miR-376a-3p |  |
| HeLa | hsa-miR-376a-3p |  |
| MCF-7 | hsa-miR-376b-3p |  |
| iMN | mmu-miR-376b-3p |  |
| MEF | mmu-miR-376b-3p |  |
| iMN | mmu-miR-409-3p |  |
| MEF | mmu-miR-425-5p |  |
| iMN | mmu-miR-431-5p |  |
| MEF | mmu-miR-450b-5p |  |
| MEF | mmu-miR-485-3p |  |
| iMN | mmu-miR-495-3p |  |
| MEF | mmu-miR-495-3p |  |
| MEF | mmu-miR-503-5p |  |
| MEF | mmu-miR-532-5p |  |
| A549 | hsa-miR-543-3p |  |
| HeLa | hsa-miR-543-3p |  |
| iMN | mmu-miR-543-3p |  |
| MEF | mmu-miR-543-3p |  |
| MCF-7 | hsa-miR-652-5p |  |
| iMN | mmu-miR-665-3p |  |
| MEF | mmu-miR-665-3p |  |
| iMN | mmu-miR-672-5p |  |
| MEF | mmu-miR-672-5p |  |
| iMN | mmu-miR-744-5p |  |
| S2 | dme-miR-996-3p |  |
| S2 | dme-miR-999-3p |  |
| S2 | dme-miR-1002-5p |  |
| S2 | dme-miR-1012-3p |  |
| MCF-7 | hsa-miR-1247-5p |  |
| MEF | mmu-miR-1247-5p |  |
| *Added* | | |
| MCF-7 | hsa-miR-20a-5p | Also found in MEF. |
| MEF | mmu-miR-193b-3p | Family member also found in MEF. |
| MEF | mmu-miR-369-3p |  |
| MEF | mmu-miR-409-3p | Also found in iMN. |
| *Removed* | | |
| K562 (KO) | hsa-miR-7-5p | Also found in multiple other systems. |
| iMN | mmu-miR-92a-3p | Also found in MEF. |
| iMN | mmu-miR-297c-5p |  |

**Table S2.** Raw initial parameter values for BBUM model fitting.

| Initial value set | *λ* | *a* | *θ* | *r* |
| --- | --- | --- | --- | --- |
| 1 | 0.9 | 0.9 | 0.1 | 0.1 |
| 2 | 0.9 | 0.1 | 0.9 | 0.1 |
| 3 | 0.9 | 0.1 | 0.1 | 0.9 |
| 4 | 0.1 | 0.1 | 0.9 | 0.9 |
| 5 | 0.1 | 0.9 | 0.1 | 0.9 |
| 6 | 0.1 | 0.9 | 0.9 | 0.1 |

**
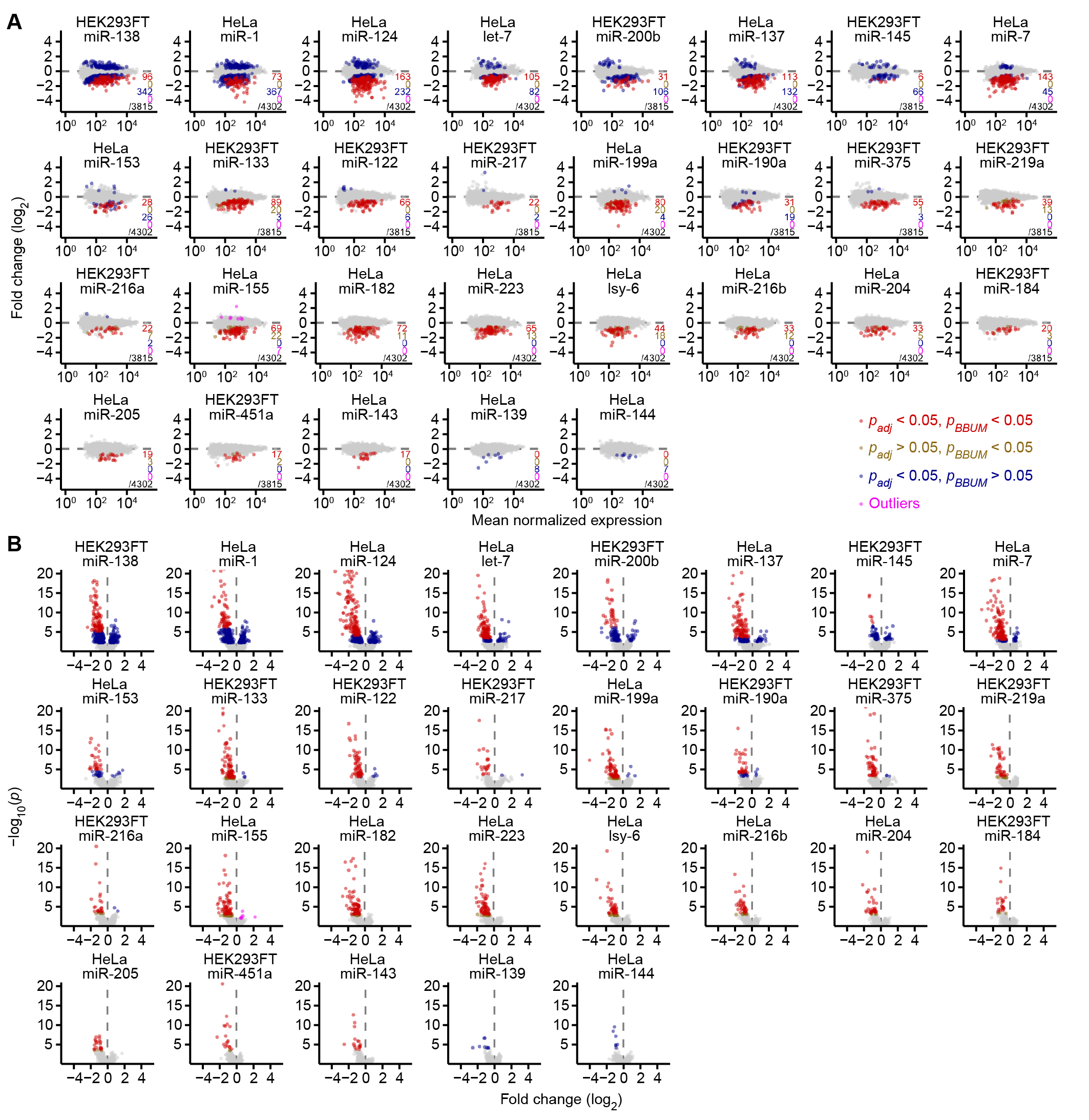
Supplementary Figure S1.** Application of the BBUM correction method to RNA-seq datasets measuring the effects of transfecting a miRNA into either HeLa or HEK293FT cells. **(A)** Plots of log_2_ fold changes in mRNA levels observed upon transfection of the indicated miRNA duplexes, showing all 29 datasets analyzed. Colors are as in Figure 5. **(B)** Volcano plots of raw *p* values as a function of mRNA log_2_ fold change, showing all 29 datasets analyzed. Colors are as in Figure 5.
